# Simultaneous quantitative respirometry and fluorometric assays in dissected hippocampal tissue from mice

**DOI:** 10.1016/j.xpro.2024.102988

**Published:** 2024-04-16

**Authors:** Sreemathi Logan, Rojina Ranjit, Hadyn Rose, Anne Bredegaard, Carlos Manlio Díaz-García

**Affiliations:** 1 Department of Biochemistry and Physiology, University of Oklahoma Health Sciences Center, Oklahoma City, OK, USA; 2 Center for Geroscience and Healthy Brain Aging, University of Oklahoma Health Sciences Center, Oklahoma City, OK, USA; 3 Stephenson Cancer Center, University of Oklahoma Health Sciences Center, Oklahoma City, OK, USA; 4 Harold Hamm Diabetes Center, University of Oklahoma Health Sciences Center, Oklahoma City, OK, USA

**Keywords:** Metabolism, Molecular Biology, Neuroscience

## Abstract

Respirometry is a technique for studying mitochondrial function that has proven compatibility with ≥0.5 mg of brain tissue. Here, we present a protocol for assessing oxygen consumption and H_2_O_2_ production rates in hippocampal tissue using the Oroboros O2k system. We describe steps for brain harvesting, tissue preparation, hippocampal microdissection, and respirometry assays. This approach has been valuable to study the metabolism of dentate granule cells of the hippocampus and could be applicable to other brain subregions.

For complete details on the use and execution of this protocol, please refer to Rose et al.^1^

## Highlights

• Microdissection of the dentate gyrus from the mouse hippocampus and mass calculation
• Permeabilization of bulk or microdissected hippocampal tissue
• Measurement of oxygen consumption rate and H_2_O_2_ production in small tissue samples

## Abstract (editor highlights)

Publisher’s note: Undertaking any experimental protocol requires adherence to local institutional guidelines for laboratory safety and ethics.

## Graphical abstract

**Figure:**
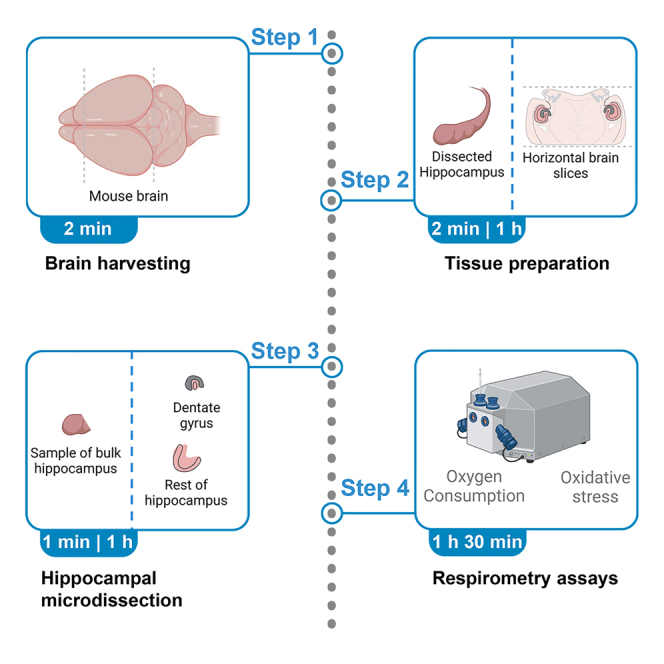

## Teaser

Respirometry is a technique for studying mitochondrial function which has proven compatibility with ≥0.5 mg of brain tissue. Here, we present a protocol for assessing oxygen consumption and H_2_O_2_ production rates in hippocampal tissue using the Oroboros O2k system. We describe steps for brain harvesting, tissue preparation, hippocampal microdissection, and respirometry assays. This approach has been valuable to study the metabolism of dentate granule cells of the hippocampus and could be applicable to other brain subregions.

## Before you begin

Here we provide a detailed protocol for the simultaneous monitoring of oxygen consumption and H_2_O_2_ production in small samples (0.5–2 mg) from the mouse hippocampus. We have successfully used the assessment of O_2_ consumption in hippocampal tissue to study how cellular metabolism impacts cognitive decline.^1^^,^^2^

Mitochondria are highly dynamic organelles that are crucial for energy metabolism. They can also act as a calcium sink during stimulation, and as a source of reactive oxygen species.^3^ Physiological changes in mitochondria are important for neurodevelopment and neurotransmission, but de-regulation of its functions can lead to altered synaptic plasticity, reduced dendritic and axonal projections, or even neuronal loss.^4^ Defective mitochondrial function in other neural cells, like astrocytes, can also lead to defective respiration and/or exacerbated production of reactive oxygen species,^5^ both of which have been linked to cognitive aging.^6^

This methodology is compatible with hippocampal tissue collected immediately after mouse euthanasia, or after typical slicing procedures used for electrophysiology and imaging experiments.

### Institutional permissions

All experiments must be performed in compliance with national and institutional regulations. The procedures here described comply with the NIH Guide for the Care and Use of Laboratory Animals and the Animal Welfare Act, and were approved by, and followed the guidelines of, the Institutional Animal Care and Use Committee of the University of Oklahoma Health Sciences Center.

Experimenters must receive similar permissions from their corresponding institutions to conduct the procedures described below.

### Preparing for tissue collection - modality 1: bulk hippocampus

**Timing: 2 min in total**

The steps described below correspond to the collection of bulk hippocampal tissue.

1. Autoclave surgical tools (scissors, scalpel, forceps, etc.).
2. Prepare buffers A, B and X, Z, saponin and wash buffer.
3. Cover bench area with absorbent surgical pads.
4. Assemble metal block on ice bucket.
5. Place weigh paper on metal block, lightly wet with ice to allow it to stick to metal block.
6. Set up a beaker with 100% ethanol for cleaning surgical instruments for use between animals.
7. Proceed to tissue collection from mouse.

### Preparing for tissue collection - modality 2: acute hippocampal slices

**Timing: 2 h in total**

The steps described below are not a continuation of the previous procedure, but an alternative sample preparation.

8. Prepare sucrose-based slicing solution the day before the experiments and keep at 4°C until use.
9. Prepare artificial cerebrospinal fluid (ACSF) solution without glucose and keep at 4°C until use. Add glucose on the day of the experiments.
10. Make a recovery chamber for slices:
  a. In a 6 × 6 × 5.5 cm plastic container, attach a tube to the bottom of the chamber, starting at the center and exiting in one of the upper corners.
  b. Take a plastic grid of 5 × 5 cm, divided in 9 wells of 1.5 cm side, and cover the bottom with a mesh.
  c. Remove the area covering the well in the center.
  d. Insert the grid in the recovery chamber with the mesh facing down.
11. Make sure that a supply of carbogen 95% O_2_ / 5% CO_2_ is available and with multiple lines to connect.
12. In the bench area used for dissections, install a water bath, a heat block, and the Compresstome (vibrating tissue slicer).
13. Keep the Compresstome accessories nearby (chilling block, blade holder with blade, specimen block).
14. Locate a clean set of the following instruments: spatula, #10 scalpel, dissecting scissors, and fine forceps.
15. Keep a transfer pipette (7.5 mL), a similar transfer pipette cut at the 1 mL level, a tube of Krazy glue, and a piece of filter paper nearby.
16. Place a clean 250 mL beaker and two 50 mL beakers in the area.
17. Prepare a 2% agarose solution in PBS and aliquot in 2 mL Eppendorf tubes. Leave at room temperature until use.
18. Find ice buckets that can fit the beakers and the Compresstome chilling block.
19. In a fume hood, place a 2 L beaker and cover the bottom with paper towels. Have a cover made of aluminum foil ready for use.
20. Take a 50 mL Falcon tube and poke (or drill) holes in the walls. Put a piece of paper on its bottom and place it inside the 2 L beaker.
21. In the fume hood, place a small bag for biological waste.

## Key resources table

**Table:** 

REAGENT or RESOURCE	SOURCE	IDENTIFIER
**Chemicals, peptides, and recombinant proteins**		

Ethylene glycol-bis(2-aminoethylether)-*N,N,N′,N′*-tetraacetic acid (EGTA)	Sigma	Cat#E4378
Creatine monohydrate	Sigma	Cat#C3630
Saponin	Sigma	Cat#S4521
Dimethyl sulfoxide (DMSO)	Sigma	Cat#D8418
Imidazole	Sigma	Cat#I2399
Taurine	Sigma	Cat#T8691
Adenosine 5′-triphosphate disodium salt hydrate (ATP)	Sigma	Cat#A6419
Phosphocreatine disodium salt hydrate (PCr)	Sigma	Cat#P7936
Magnesium chloride hexahydrate (MgCl_2_· 6H_2_O)	Sigma	Cat#M2670
Potassium hydroxide (KOH)	Sigma	Cat#221473
MES potassium salt (K-MES)	Sigma	Cat#M0895
Potassium chloride (KCl)	Sigma	Cat#P9541
Potassium phosphate dibasic (KH_2_PO_4_)	Sigma	Cat#P3786
Bovine serum albumin (BSA)	Sigma	Cat#A3803
L-Glutamic acid, sodium salt-hydrate (C_5_H_8_NO_4_Na)	Sigma	Cat#G5889
L-Malic acid (C_3_H_4_O_4_)	Sigma	Cat#M7397
Sodium pyruvate	Sigma	Cat#P2256
Adenosine 5′diphosphate monopotassium salt (ADP)	Sigma	Cat#A5285
Ascorbate sodium salt (C_6_H_7_O_6_Na)	Sigma	Cat#A7631
N,N,N′,N′-Tetramethyl-p-phenylenediamine dihydrochloride (TMPD; C_10_H_15_N_5_O_10_P_2_K)	Sigma	Cat#T3134
Cytochrome *c*	Sigma	Cat#C2506
Rotenone (C_23_H_22_O_6_)	Sigma	Cat#R8875
Superoxide dismutase (SOD)	Sigma	Cat#S8160
Antimycin A (AA)	Sigma	Cat#A8674
Amplex UltraRed	Invitrogen	Cat#A36006
Horseradish peroxidase (HRP)	Sigma	Cat#P8375
Hydrogen peroxide solution (H_2_O_2_)	Sigma	Cat#H1009
200 proof ethanol	Koptec	Cat#64-17-5
Succinate	Sigma	Cat#S2378
Calcium carbonate (CaCO_3_)	Sigma	Cat#239216

**Experimental models: Organisms/strains**		

Mouse: C57BL/6J (6–12 months) male mice	The Jackson Laboratory	RRID: IMSR_JAX:000664

**Software and algorithms**		

ImageJ 1.52a	Schneider et al.^7^	https://imagej.nih.gov/ij/
DatLab version 7.4.0.4	Oroboros	https://www.oroboros.at/?s=datlab

**Other**		

Balance	Mettler Toledo	Cat#XS105
Microsyringe\25 mm^3^ 51/0.15 mm	Oroboros	Cat#51025-01
Microsyringe\10 mm^3^ 51/0.13 mm	Oroboros	Cat#51010-01

## Materials and equipment

**Table:** Slicing Solution

Reagent	Final concentration	Amount
NaCl	87 mM	5.08 g
KCl	2.5 mM	0.19 g
NaH_2_PO_4_· H_2_O	1.25 mM	0.17 g
NaHCO_3_	25 mM	2.10 g
CaCl_2_ · 2H_2_O	0.5 mM	0.07 g
MgCl_2_	7 mM	7 mL (of 1 M stock)
Sucrose	75 mM	25.67 g
D-Glucose	25 mM	4.50 g
ddH_2_O	N/A	To 1 L
**Total**	**N/A**	**1 L**

Dissolve in 800 mL double-distilled water and adjust to 1 L. This buffer can be stored at 4°C for two weeks.

**Table:** Artificial Cerebrospinal Fluid (ACSF)

Reagent	Final concentration	Amount
NaCl	125 mM	7.31 g
KCl	2.5 mM	0.19 g
NaH_2_PO_4_ · H_2_O	1 mM	0.14 g
NaHCO_3_	26 mM	2.18 g
CaCl_2_ · 2H_2_O	2.5 mM	0.37 g
MgCl_2_	1.2 mM	1.2 mL (of 1 M stock)
D-Glucose	5 mM	0.90 g
ddH_2_O	N/A	To 1 L
**Total**	**N/A**	**1 L**

Dissolve in 800 mL double-distilled water and adjust to 1 L. This buffer can be stored at 4°C for two weeks. Add glucose on the day of the experiments.

**Table:** Buffer A

Reagent	Final concentration	Amount
Ethylene glycol-bis(2-aminoethylether)-*N,N,N′,N′*-tetraacetic acid (EGTA)	100 mM	7.61 g
Potassium hydroxide (KOH)	200 mM	2.24 g
ddH_2_O	N/A	To 200 mL
**Total**	**N/A**	**200 mL**

Adjust solution pH to 7.5 with KOH.

**Table:** Buffer B

Reagent	Final concentration	Amount
Calcium carbonate (CaCO_3_)	100 mM	2.002 g
Buffer A	N/A	To 200 mL
**Total**	**N/A**	**200 mL**

Dissolve the CaCO_3_ in Buffer A at 80°C solution while stirring continuously and adjust pH to 7.5 with KOH.

**Table:** Buffer X

Reagent	Final concentration	Amount
Buffer A	N/A	36.15 mL
Buffer B	N/A	13.85 mL
Imidazole	20 mM	0.68 g
DTT	0.5 mM	0.0386 g
Taurine	20 mM	1.26 g
ATP	5.7 mM	1.57 g
Phosphocreatine disodium salt hydrate (PCr)	14.3 mM	1.82 g
MgCl_2_–6H_2_O	6.56 mM	0.68 g
K–MES	50 mM	5.83 g
ddH_2_O	N/A	To 1 L
**Total**	**N/A**	**1 L**

Dissolve in 800 mL double-distilled water and adjust to pH of 7.1 with 5 M hydrochloric acid (a few drops) and bring up to final volume to 1 L. This buffer can be stored at −20°C frozen. Store in 50 mL aliquots. Once thawed, use within a week.

**Table:** Buffer Z

Reagent	Final concentration	Amount
K–MES	105 mM	12.26 g
KCl	30 mM	1.12 g
KH_2_PO_4_	10 mM	0.7 g
MgCl_2_· 6H_2_O	5 mM	0.51 g
Bovine Serum Albumin	0.5 mg/mL	0.25 g
ddH_2_O	N/A	To 1 L
**Total**	**N/A**	**1 L**

Dissolve in 800 mL double-distilled water and adjust to pH of 7.1 and bring up to final volume to 1 L. This buffer can be stored at −20°C frozen. Store in 50 mL aliquots. Once thawed, use within a week.

**Table:** Assay Buffer

Reagent	Final concentration	Amount
Creatinine monohydrate	20 mM	29.84 mg
EGTA	1 mM	100 μL (of 100 mM stock)
Buffer Z	N/A	10 mL
SOD	5 U/mL	25 μL (of 2000 U/mL stock)
HRP	1 U/mL	33.3 μL (of 300 U/mL stock)
Amplex-Red	10 μM	10 μL (of 10 mM stock)
**Total**	**N/A**	**10 mL**

Dissolve creatinine monohydrate and EGTA in buffer Z at 37°C for 15–30 min and then add the other components (SOD, HRP and Amplex-Red) of Assay buffer. Prepare fresh Assay buffer on the day of experiments. Store the assay at 4°C buffer protected from light.

• **Wash buffer**: 1 mM EGTA in buffer Z (mix 500 μL of 100 mM stock of EGTA in 50 mL of buffer Z).

Store at 4°C for up to 7 days.

• **1% Saponin [10 mg/mL]:** 0.01 g Saponin in 1 mL of ddH_2_O.

Mix by rotation at 4°C for 3 min (∗freshly prepared prior to use) –prepare 1 mL per day. Store on ice.

## Step-by-step method details

### Tissue collection - modality 1: bulk tissue collection from the mouse hippocampus

**Timing: 2 min in total**

The following steps detail the procedure for dissecting the hippocampus from whole brain tissue.

1. Following cervical dislocation and decapitation, rapidly remove the brain from the skull, and place it on the ice-cold block with weigh paper.

**CRITICAL:** Do not place brain tissue directly on metal block as this would cause tissue to stick to the metal leading to poor recovery of sample.

2. Cut the brain along the midline and remove midbrain structures.
3. Roll out hippocampus and immediately place it in buffer X in a weigh boat on ice (∼less than 1 min from cervical dislocation).
4. Dissect a small piece of dorsal hippocampus and partially dry with a delicate task wiper (e.g., Kimwipes).
5. Weigh the hippocampus in the analytical balance. The dissected piece should ideally be between 2–3 mg.
6. Transfer this tissue into a 2 mL Eppendorf tube with 1.5 mL ice-cold buffer X and proceed with the respirometry assays (Step 91).

### Tissue collection - modality 2: dissection of dentate gyri from acute hippocampal slices

**Timing: 3 h**

The following steps describe the preparation of acute brain slices and the microdissection of the dentate gyrus from the rest of the hippocampus.

7. Turn on the water bath and set the temperature at 37°C.
8. Mount the recovery chamber.
9. Add ACSF solution with glucose until it covers the insert.
10. Connect it to the carbogen (95% O_2_ and 5% CO_2_) supply.
11. Begin bubbling and cover the chamber with a 100 mm Petri dish or aluminum foil.

***Note:*** The bubbling provides oxygenation and ensures a stable pH ∼7.4 in the solution.

12. Place the chamber inside the water bath while continuously oxygenating the ACSF.
13. Turn on the heat block and set the temperature at 65°C.
14. Insert a 2 mL Eppendorf tube containing PBS with 2% agarose until it melts.
15. Set the heat block to 42°C and keep the agarose-PBS mix at this temperature until used.
16. Fill an ice bucket with ice and place a 250 mL beaker on it.
17. Add ∼250 mL of ice-cold slicing solution in the large beaker.
18. Insert a gas dispersion tube connected to the carbogen supply and bubble the solution continuously.
19. Fill another ice-bucket with ice and place a 50 mL beaker on it.
20. Add 20–30 mL of ice-cold slicing solution to the 50 mL beaker.
21. Insert a P200 pipette tip connected to a carbogen line and bubble with carbogen continuously.
22. Insert the chilling block of the Compresstome into the ice.
23. Make sure that all necessary accessories are nearby (specimen tube, blade holder with cutting blade, and Allen wrench to secure the blade holder to the instrument).
24. Add ice-cold slicing solution into another 50 mL beaker and take it to the fume hood.
25. In the fume hood, pipette 250 μL of isoflurane into the conical tube with holes and immediately place it into a 2 L beaker.
26. Transfer the mouse into the 2 L beaker.
27. Cover the 2 L beaker with aluminum foil.
28. Monitor the induction of anesthesia.
29. Check for deep anesthesia periodically by pinching the paw or tail.

***Note:*** Return the animal inside the beaker if still responsive. If unresponsive, proceed with the next steps.

30. Using sharp, heavy-duty scissors, quickly decapitate the mouse and submerge the head in the 50 mL beaker with slicing solution.
31. Quickly move to the dissection area.
32. Using fine scissors, cut the scalp open in a caudal to rostral direction, to expose the skull.
33. If necessary, remove muscles from the neck with a transverse cut below the occipital bone.
34. Using dissection scissors, cut the skull through the sagittal suture (midline), in a caudal to rostral direction (Figure 1).

**Figure 1:**
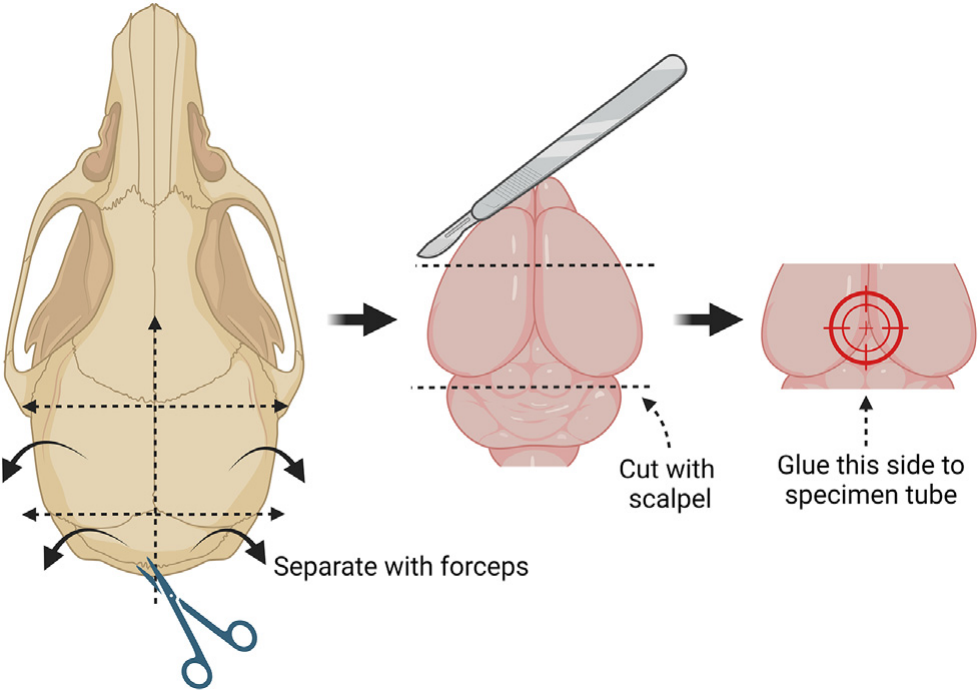
Brain dissection prior to the preparation of acute hippocampal slices The sequence begins with accessing the brain by cutting through the mouse skull using dissecting scissors. The direction of the cuts is represented by dashed arrows. The resulting sections are then separated using fine forceps. Once the brain is exposed, cut the tissue using a scalpel and gently scoop the tissue into oxygenated slicing solution. Finally, apply glue on the specimen tube and deposit the brain by its dorsal side.
35. Make horizontal cuts near bregma and lambda in both directions along the sutures. This will create several flaps that can be pulled back using forceps.
36. Once the brain is exposed, make a blunt cut with the scalpel in the coronal plane just above the cerebellum and pull away this region.
37. Perform a second cut in the frontal lobe and pull away the tissue located rostrally.
38. Use the spatula to gently remove the brain and place it in the 50 mL beaker with the slicing solution under continuous bubbling.

**CRITICAL:** It is important to minimize the time that the brain remains without oxygenation to avoid tissue damage.

39. Put a drop of glue onto the cylinder of the specimen tube.
40. Collect the brain with the spatula again and remove the excess of solution with the filter paper.
41. Gently slide the brain onto the solid cylinder of the specimen tube, ensuring that it becomes glued on the dorsal side.
42. Remove the Eppendorf tube with agar from the heat-block and collect the contents with a transfer pipette.
43. Move the metallic outer cylinder of the specimen tube up and add the molten 2% agar solution onto the brain inside it.
44. Quickly insert the specimen tube into the chilling block and hold the block handles tightly.

***Note:*** The agar’s appearance becoming opaque will indicate that it has solidified.

45. Place the cylinder into the slicing tray of the Compresstome until the hilt of the metal part of the cylinder is all the way to the tray.

***Note:*** Be careful not to press the white cylinder through the metal.

46. Pour the slicing solution from the 250 mL beaker into the Compresstome tray until the cylinder is covered.
47. Slide the tray onto the instrument and secure it with a screw.
48. Put the blade holder with a blade on and secure it to the instrument using an Allen wrench.
49. Move the metal rod of the instrument until it meets with the cylinder in the specimen tube.
50. Continue moving the rod forward until the agar moves out of the cylinder far enough to visualize the edge of the brain.
51. Set the thickness of the tissue sections at 400 μm and begin slicing.

***Note:*** Brain slices can be prepared with different solutions and procedures, as previously reported.^8^^,^^7^

52. Immediately after slicing the brain, use a transfer pipette (with a larger tip opening) to move the slices to a 100 mm Petri dish containing ice-cold slicing solution.

***Note:*** Keep the solution bubbled with carbogen.

**Figure 2:**
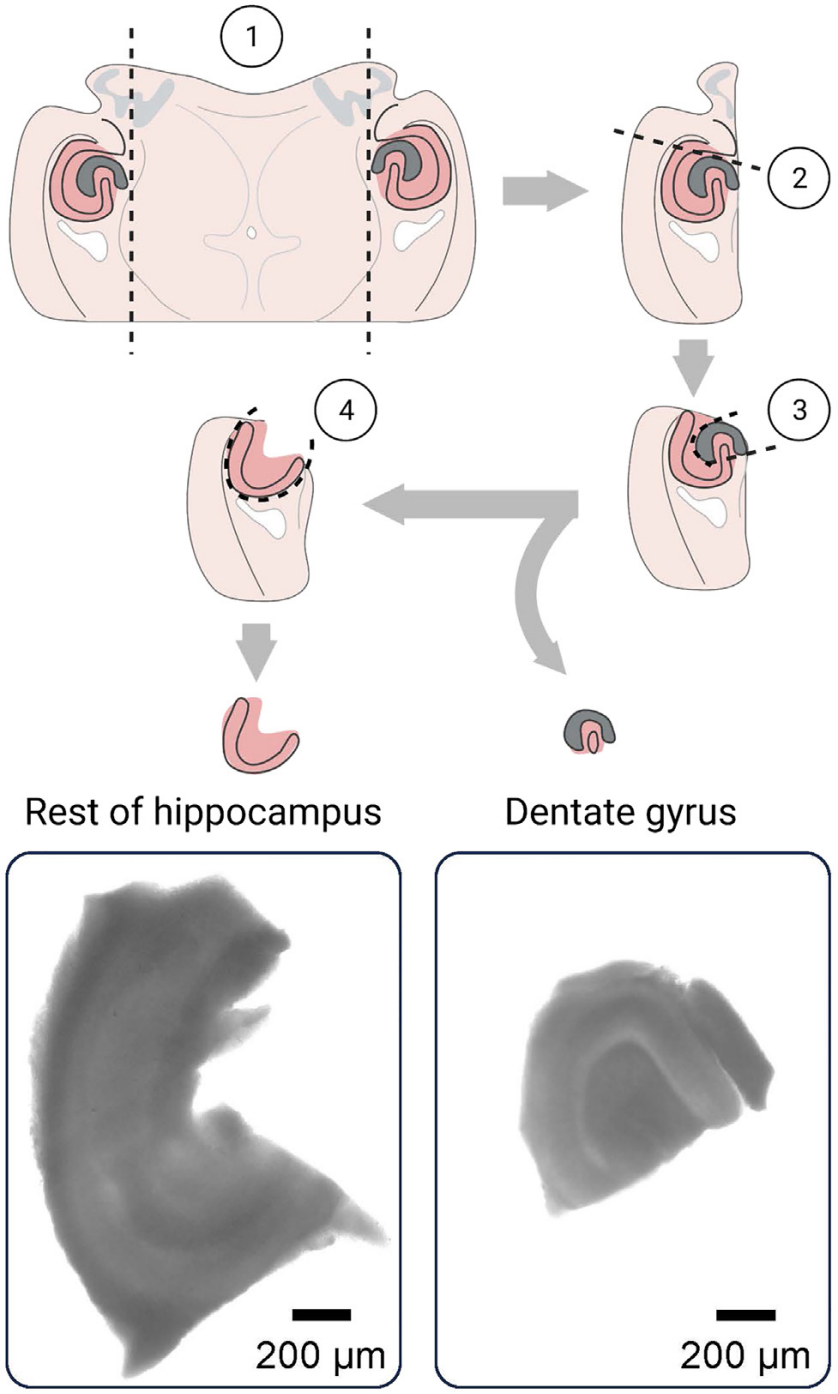
Dissection of hippocampal subregions for respirometry analysis Graphics depict a horizontal brain slice and the successive cuts to obtain the dentate gyrus and the rest of the hippocampal tissue. During the procedure, the tissue can be held, or pressed against the bottom of the Petri dish, using forceps in other areas of the brain slice while avoiding damage to the hippocampus. We suggest progressively (and gently) cutting and separating the tissue in steps 3 and 4. Images at the bottom correspond to preparations used for experiments in the O2k instrument.

***Note:*** During these manipulations, the slice can be held in place with the Dumont # 5 forceps pinching on other brain areas.

53. Trim the resulting horizontal slices near the hippocampus in both hemispheres, using a #10 scalpel. Follow the anterior-posterior axis, as depicted in the first step of Figure 2.

54. While visualizing one of the hemispheres under a dissecting stereoscope, perform an initial cut tangential to the dentate gyrus and through the subiculum and the cortex (see step 2 in Figure 2).

***Note:*** The steps below are meant as a blueprint, but experimenters are encouraged to try variations of these cuts if that improves the time to complete the procedure.

55. Complete the dissection of the dentate gyrus by making an incision through the imaginary line connecting the extremes of the suprapyramidal and infrapyramidal blades.
56. Make a tangential cut to the molecular layer and continue advancing with small cuts around the perimeter of the dentate gyrus.

***Note:*** Gently separate the rest of the hippocampus with the scalpel.

57. Finally, cut through the fimbria and continue along the alveus until the rest of the hippocampus is fully separated from the slice of brain tissue.
58. Collect the tissue with a 7.5 mL transfer pipette (widened in the tip after a blunt cut) and place it on the recovery chamber filled with artificial cerebrospinal fluid (ACSF) at 37°C.

**CRITICAL:** Keep the solution bubbled with carbogen.

***Note:*** Slices can also be retrieved using P200 or P1000 pipettes with wider tips after a blunt cut, to allow unimpaired movement of the tissue.

59. Incubate the slices at 37°C for 35 min, then place them at room temperature until used for respirometry.

***Note:*** Use the slices within 2 h after the recovery period.

60. Take two dissected dentate gyri, or a hippocampal slice without the dentate gyrus, and place them in a 35 mm Petri dish containing pre-oxygenated ACSF.
61. Immediately take pictures of these samples using transmitted light or differential interference contrast-infrared illumination. Make sure to image the entire dissected slices.

***Note:*** We obtained the images with a Nikon Eclipse Ti-U microscope. The goal is to obtain a calibrated measurement of the slice contours (and visual confirmation of the anatomical landmarks of the preparation if possible).

**CRITICAL:** If the image dimensions are not automatically saved, make sure to annotate the image resolution in pixels, and the pixel size in microns.

62. For the determination of tissue mass in microdissected slices, open an image encompassing the whole area of the slice of interest (e.g., dissected dentate gyrus) using the ImageJ software.^9^

***Note:*** Steps described below are based on software version ImageJ 1.52a. There might be slight variations in other versions.

63. Click on the “Analyze” button in the main panel, a dropdown menu will appear.
  a. Click on “Select Measurements…”
  b. Select the option “Area” and de-select any other pre-selected options.
  c. Confirm your choices by clicking the “OK” button.
    ***Note:*** If the image dimensions in microns are not read automatically, click on “Analyze” in the main menu and:
  d. Select “Set Scale…” from the dropdown menu.
  e. Then introduce the distance in pixels and microns in the corresponding boxes.
  f. Confirm the selections by clicking the “OK” button.
64. Click on the “Polygon selections” button in the main menu and draw a region of interest following the contours of the slice of interest.
65. Click on “Analyze” in the main menu.
  a. Select “Tools” from the dropdown menu, an additional lateral dropdown menu will appear.
  b. Select “ROI Manager…” from this list.
  c. In the “ROI Manager” pop-up box, click the button “Add [t]” to include the region of interest for further analysis.

***Note:*** It is strongly recommended to save this region of interest for future reference by clicking the button “More >>”, then “Save”.

66. Click on the “Measure” button to obtain the area of the slice in squared microns.
67. To calculate the volume of the slice, multiply the area by the thickness of the slice.

***Note:*** In our experiments, the slice thickness was set at 400 μm in the vibrating microtome.

68. To estimate the mass of the slice, multiply the volume by the reported brain tissue density of 1.081 g/cm^3^, as reported by Barber et al. (1970).^10^

***Note:*** Prior to any experiments, we validated this approach by measuring five dentate gyri slices with the procedure described above, which resulted in an estimated mass of 0.27 ± 0.05 mg per slice (aggregated mass = 1.34 mg).

The same slices were pooled together and weighed by another investigator (who was blind to the initial calculations) in an analytical balance. The weight of the pooled slices was 1.36 mg (within a ∼2% difference from our estimations), corresponding to an average weight for individual slices of ∼0.27 mg, in agreement with our estimations.

69. Transfer the slices into a 2 mL Eppendorf tube with 1.5 mL ice-cold buffer X and proceed with the respirometry assays.

### Respirometry analysis using the O2k system

#### Running H_2_O_2_ standards

**Timing: 30 min**

The following steps are essential for the calibration of the O2k system for the measurement of reactive oxygen species (ROS) quantification in O2k fluorometry. A standard curve for H_2_O_2_ is necessary for accurate detection of hydrogen peroxide within the brain tissue.

70. Connect the Smart Fluo-Sensor cable (Green LED) to the Fluo plug and turn on O2k equipment.
71. Initiate ‘Oroboros DatLab’ to start the program and connect to O2k.

***Note:*** Set up of Oroboros O2k chambers, software usage should be followed as specified by the manufacturer and available at https://wiki.oroboros.at/index.php/MitoPedia:_DatLab

72. Assign label 1 (Power-O2k or P-number) and enter the Oxygen sensor number, which can be found on the side of the POS for both chambers A and B.
73. Set the Block temperature to 37°C and stirrer speed to 750 rpm and the data recording interval to 2 s.
74. In the Oxygen tab of the software, set Gain of 1 and the polarization voltage to 800 mV.
75. In the Amperometric tab, set the Gain for Amp sensor to 1000 and the Amp polarization voltage to 500 mV.
76. Add ∼2.1 mL of assay buffer to each chamber and place the stopper down slowly.

***Note:*** As HRP and Amplex-Red are light-sensitive, care should be taken to keep in dark environment. Chamber illumination should be switched off.

**Figure 3:**
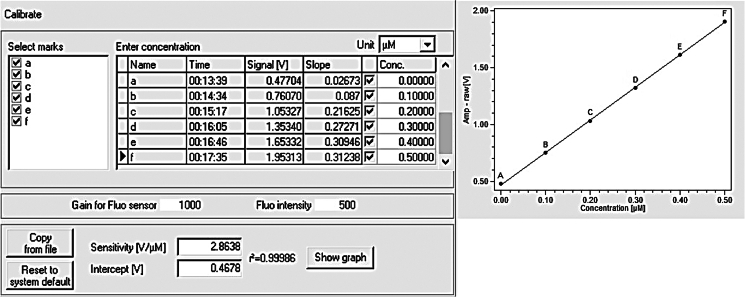
Calibration curve of the H_2_O_2_ signal The figure shows the software layout: a table with typical values, and the selection of parameters for the experiment. The resulting calibration curve is illustrated to the right with an r^2^ = 0.99986.

***Note:*** This will generate a calibration curve for accurate measurements of H_2_O_2_ in brain tissue.

77. Assign experiment name, enter sample ID, and ensure chamber volume is set at 2 mL.
78. On the ‘Layout’ menu on top of the screen, select “O2&Amp” then “Standard layout” then “01 Amp Amperometric Raw signal”.
79. To generate a standard curve, get baseline recording.
80. Using a clean syringe (washed 3× with ddH_2_O), inject 0.1 μM (5 μL of 40 μM H_2_O_2_ stock in chamber volume of 2 mL) of H_2_O_2_.
81. Wait for Amp Signal to stabilize and then select the plot for 'H_2_O_2_ raw' and mark a brief section immediately before injection for baseline reading and after the addition of H_2_O_2_.
82. Repeat the 5 μL of 40 μM H_2_O_2_ injection four more times to generate curve of 0.2, 0.3, 0.4, 0.5 μM of H_2_O_2_.
83. Mark each concentration’s corresponding slope on DatLab (Figure 3), which confirms the signal sensitivity increase ([V/μM]).

84. Click on calibrate to save the data and close the window.
85. Wash the chamber 3× with ddH_2_O.

### Air calibration

**Timing: 10–15 min**

These steps allow for calibration of oxygen levels within the chambers of the O2k system, which will ensure that fluctuation and consumption of oxygen concentration from changes in mitochondrial metabolism are accurately measured.

86. Open a new file for air calibration.
87. Fill up the 2 mL chamber with 2.1 mL Assay buffer and close the chamber placing the stopper down slowly.
88. Lift the stopper slowly and put the stopper-savers leaving a gas volume above the assay buffer for final air equilibration.
89. Calibrate the chamber once the O_2_ recording has stabilized as per O2k instructions for calibration.
90. Close the chamber by pushing the stopper all the way in after recording air calibration and wait for signals to be stable again.

Troubleshooting 1.

**CRITICAL:** Air Calibration should be run before any experiment for the day to account for changes in barometric pressure or to accommodate changes in experimental temperature or medium.

***Note:*** Following Air Calibration, immediately proceed to Respirometry measurements.

### Simultaneous measurements of oxygen consumption rates and the production of reactive oxygen species

**Timing: ∼45 min**

The following procedures allow for respirometry in hippocampal tissue. Oxygen consumption measurements are made using substrates and inhibitors specific for electron transport chain proteins. These steps allow for quantification of the mitochondrial capacity for respiration during energy production.

91. Place tissue into Eppendorf tube with 1.5 mL of Buffer X and saponin (50 μg/mL).
92. Incubate at 4°C on a rotator for 30 min.
93. Wash tissue with Buffer Z three times for 5 min each at 4°C on a rotator.
94. Following washes, open chamber, place the sample in the chamber, close the chamber, and place the stopper down slowly.
95. Wait until the O_2_ slope signal stabilizes.
96. Add the following substrates and inhibitors in sequential order and record using DatLab software.

***Note:*** Wait for oxygen consumption stabilization between each substrate and inhibitor.

**Table:** 

Reagent	Stock concentration (dissolved in vehicle)	Add volume	Final concentration
Glutamate	2 M (ddH_2_0)	10 μL	10 mM
Malate	0.8 M (ddH_2_0)	5 μL	2 mM
Pyruvate	2 M (ddH_2_0)	5 μL	5 mM
ADP	0.5 M (ddH_2_0)	10 μL	2.5 mM
Succinate	1 M (ddH_2_0)	20 μL	10 mM
Rotenone	1 mM (Ethanol)	1 μL	0.5 μM
Antimycin A	5 mM (Ethanol)	2 μL	5 μM
Ascorbate	0.8 M (ddH_2_0)	5 μL	2 mM
TMPD	0.2 M (ddH_2_0)	5 μL	0.5 mM

Individual components are dissolved in appropriate vehicle to make stock concentrations. Stock concentrations of glutamate, malate, ADP and succinate are adjusted to a pH of 7.0 with 5 M KOH. Store stock solution aliquots for up to 6 months at −20°C.

97. Following O2k measurements, wash the full chamber with ddH_2_O one time for 5 min.
98. Wash the full chamber with 70% ethanol 3× for 5 min each.
99. Wash for 30 min with 100% ethanol.

***Note:*** Let the stopper go naturally into the chamber for washes and aspirate any additional ddH_2_O or ethanol.

100. Fill the top of the stopper with ethanol or water and close the cap.
101. Store the chamber until the next experiment with 70% ethanol
102. Rinse the chamber and the stopper 3× with double distilled water (ddH_2_O) for the next experiment to make sure you remove all the ethanol.

Troubleshooting 1.

## Expected outcomes

**Figure 4:**
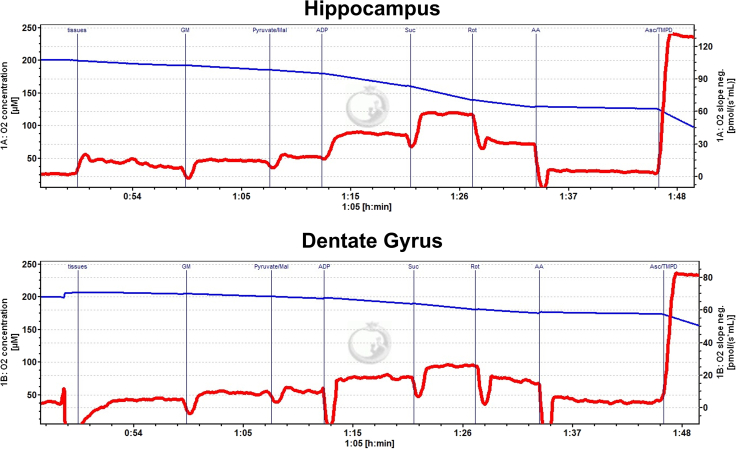
Representative traces of oxygen consumption with ETC substrates and inhibitors in isolated dorsal hippocampus and dentate tissue in the Oroboros Following addition of the tissue within the chamber and a stable red trace depicting oxygen consumption (right y-axis) is achieved, subsequent additions of substrates and ETC inhibitors are recorded (black vertical line), and stable oxygen consumption is measured. Blue line (left y-axis) represents the oxygen concentration within the chamber.

**Figure 5:**
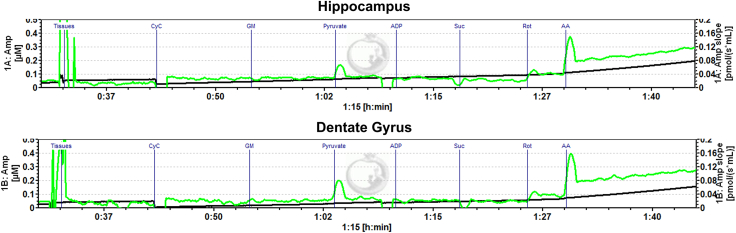
Representative traces of ROS measurements through fluorometry Hippocampal (top) and dentate (bottom) production of H_2_O_2_ is recorded for each substrate and inhibitor from Figure 2. ROS levels increase substantially in the presence of inhibitor antimycin A (AA).

**Figure 6:**
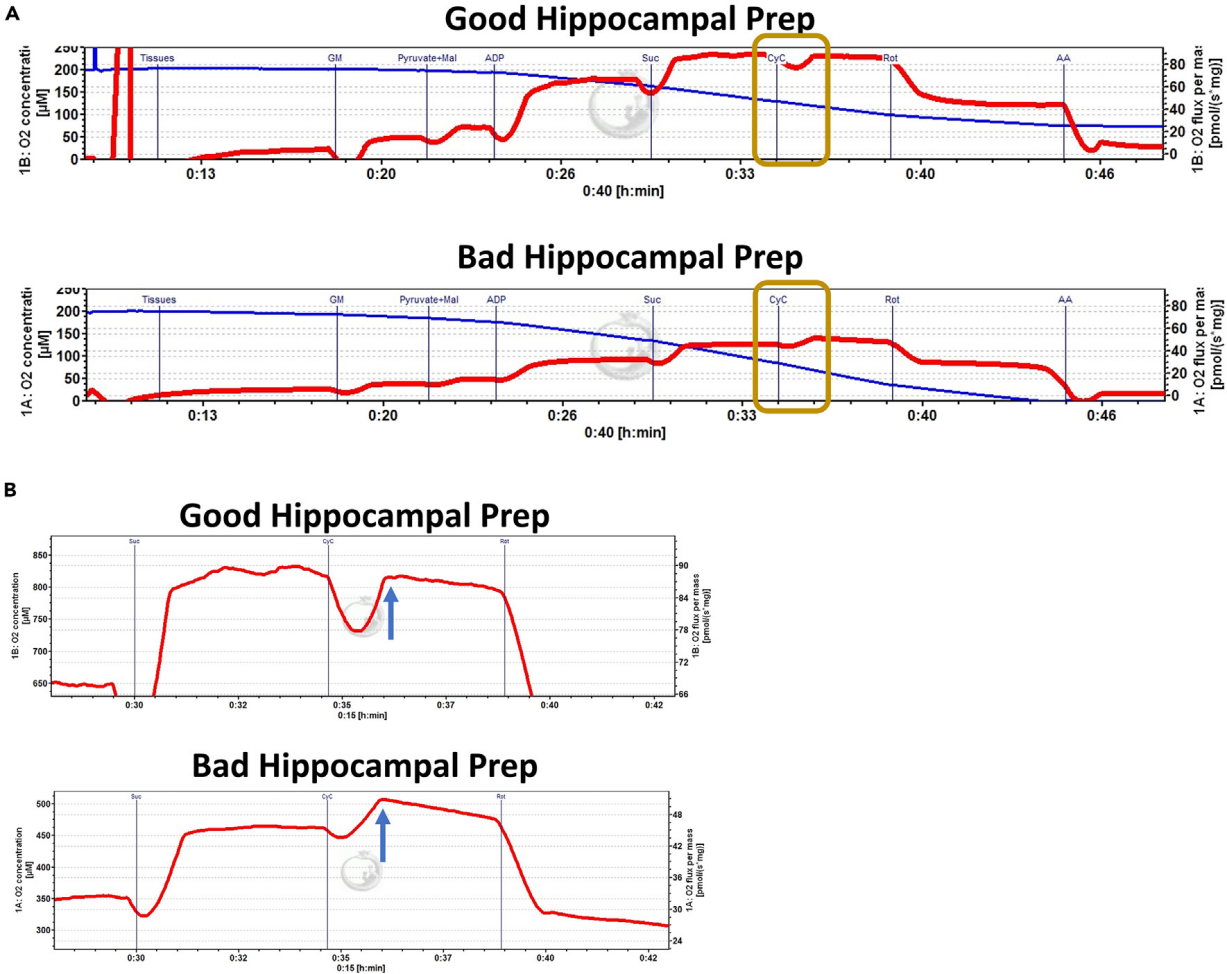
Assessing the quality of hippocampal preparations (A) Representative traces of oxygen consumption of a good hippocampal prep (top) and bad hippocampal prep (bottom). Following dissection, one hippocampus was immediately weighed and placed in buffer X, while the other hippocampus was delayed (∼10 min) before placing in the buffer to mimic damage from delayed preparations. Oxygen consumption (red) was markedly reduced in the bad prep compared to the good prep. Box around CyC measurements are magnified in B. (B) A magnified representation of the trace from each of the good and bad prep showing oxygen consumption (blue arrows) in response to cytochrome C. The bad prep (bottom) showed an increased cytochrome C induced oxygen consumption indicating damaged mitochondrial membrane, while the good prep (top) showed no change from background.

In brain tissue, we expect complex I + II respiration (after the addition of succinate) to be higher than complex I respiration alone. However, the magnitude of these changes may be different for other regions of the brain as well as other tissue types in the body. The expected results for O2K respirometry are illustrated in Figure 4 and ROS (H_2_O_2_) production in Figure 5. Quantification of respirometry and fluorometry from raw data is illustrated in Table 1 and Table 2 respectively. Cytochrome *c* (CyC) oxygen consumption indicates the integrity of the mitochondrial membrane and thereby tissue viability. To illustrate a good tissue prep from a bad prep, we delayed the timing for dissection to 10 min. An increase in oxygen consumption with CyC in the delayed prep indicates damaged/poor tissue integrity as illustrated in Figure 6.

**Table 1:** Example of OCR calculations from raw data measured by O2k respirometry

OCR (OXPHOS) calculation	Raw data pmol s^−1^ mg^−1^	OCR Corrected for non-mitochondrial respiration (Raw-AA)	Final calculated OCR pmol s^−1^ mg^−1^
**Antimycin A (AA)**	**6.3723**		
Glutamate + Malate (GM)	14.1035	14.1035–**6.3723**	7.7312
Pyruvate + Malate (PM)	23.3662	23.3662–**6.3723**	16.9939
ADP	66.3917	66.3917–**6.3723**	60.0194
Succinate (Suc)	87.7536	87.7536–**6.3723**	81.3813
Cytochrome C (CyC)	86.5884	86.5884–**6.3723**	80.2161
Rotenone (Rot)	43.4196	43.4196–**6.3723**	37.0473

**Table 2:** Example of ROS calculations from raw data measured by O2k Fluorometry

ROS calculation	Raw data (based on standard curve) pmol s^−1^ mL^−1^	ROS adjusted to chamber volume (2 mL) pmol s^−1^	ROS normalized to wet tissue weight pmol s^−1^ mg^−1^
Tissue (wet weight – 3.08 mg)			
Tissue	0.0077	0.0154	0.005
Glutamate + Malate	0.0321	0.0642	0.020844156
Pyruvate	0.0395	0.079	0.025649351
ADP	0.0168	0.0336	0.010909091
Succinate	0.0262	0.0524	0.017012987
CyC	0.0154	0.0308	0.01
Rotenone	0.0473	0.0946	0.030714286
Antimycin A	0.1027	0.2054	0.066688312

## Quantification and statistical analysis

Below is a representative table (Table 1) of respirometry measurements (OCR) from the hippocampus. Raw data acquired from the Oroboros is adjusted for non-mitochondrial respiration by subtraction of the antimycin A (AA) value to derive the final calculated OCR. Tissue weights are entered into O2k software and thus raw and final calculated OCR values are normalized to tissue weight for analysis.

Below is a representative table (Table 2) of ROS measurements from the hippocampus. Raw data acquired from the Fluorometry are derived based on the standard curve for H_2_O_2_. Raw data for ROS measurements are normalized to chamber volume (2 mL), then normalized to wet tissue weight.

## Limitations

Successful measurements of oxygen consumption are dependent on tissue integrity and size of tissue sample. The small size of the samples may impede the ability to detect their mass, which is crucial for normalization and therefore quantitation of the respirometry data. To overcome this limitation, we have estimated mass based on the volume of dentate sample derived from image analysis of slice preparations. A major limitation for normalization is also the lack of recovery of tissue after respirometry to quantify protein concentrations. This is a technical limitation that cannot be overcome due to the small size of the sample.

We expect that this protocol can be adapted to assess respirometry in other brain regions as well as other tissues across the body. However, individual parameters, such as permeabilization time, tissue size, permeabilizing reagent and substrate concentrations need to be titrated for each tissue.

## Troubleshooting

### Problem 1: No OXPHOS detected

• Lack of tissue respiration would indicate inconsistencies with reagents and or chamber oxygen levels.

### Potential solution

• Check the oxygen level; if the oxygen concentration is low inside the chamber, the sensor will not detect the accurate reading. Aerate the solution following steps for air calibration (related to steps 86–90).
• Check all the substrates and reagents and store the aliquots of stock concentration at −20°C for up to 1 year. Avoid freeze-thaw. If there is low or no OXPHOS response, then make fresh substrate and reagent (related to Step 96).

### Problem 2: High cytochrome C OXPHOS

• High CyC OXPHOS signals indicate damaged mitochondria from bad tissue preparation.

### Potential solution

• Prepare tissue dissection in appropriate time window as indicated. Increased time during dissection could result in tissue/mitochondrial damage (related to Step 1).
• If working with hippocampal slices, test a slicing solution that includes additional pH buffers and antioxidants, as previously described.^8^

### Problem 3: High background signal in OXPHOS

• Lack of tissue respiration would indicate inconsistencies with reagents and or chamber oxygen levels.

### Potential solution

• Ensure adequate levels of buffer (2 mL) inside the chamber. Always put 2.1 mL buffer in the chamber. Extra buffer will come out from the stopper (related to Step 87).
• Service the polarographic oxygen sensor (OroboPOS) in the O2k system at least twice a year.

***Note:*** The sensor’s cathode and anode need to be cleaned and the electrolyte and membrane need to be changed at least twice a year.

## Resource availability

### Lead contact

Further information and requests for resources and reagents should be directed to and will be fulfilled by the lead contact, Sreemathi Logan (sreemathi-logan@ouhsc.edu).

### Technical contact

Questions about the technical specifics of performing the protocol should be directed to and will be answered by the technical contacts, Sreemathi Logan (sreemathi-logan@ouhsc.edu) and Carlos Manlio Díaz-García (carlosmanlio-diazgarcia@ouhsc.edu).

### Materials availability

This study did not generate new unique reagents.

### Data and code availability

This study did not generate/analyze [datasets/code].
